# Cross-Language Measurement Equivalence of the Center for Epidemiologic Studies Depression (CES-D) Scale in Systemic Sclerosis: A Comparison of Canadian and Dutch Patients

**DOI:** 10.1371/journal.pone.0053923

**Published:** 2013-01-11

**Authors:** Linda Kwakkenbos, Erin Arthurs, Frank H. J. van den Hoogen, Marie Hudson, Wim G. J. M. van Lankveld, Murray Baron, Cornelia H. M. van den Ende, Brett D. Thombs, for the Canadian Scleroderma Research Group

**Affiliations:** 1 Department of Rheumatology, Sint Maartenskliniek Nijmegen, The Netherlands; 2 Department of Psychiatry, McGill University, Montréal, Québec, Canada; 3 Department of Epidemiology, Biostatistics, and Occupational Health, McGill University, Montréal, Québec, Canada; 4 Department of Medicine (Division of Rheumatology), McGill University, Montréal, Québec, Canada; 5 Department of Educational and Counselling Psychology, McGill University, Montréal, Québec, Canada; 6 School of Nursing, McGill University, Montréal, Québec, Canada; 7 Lady Davis Institute for Medical Research, Jewish General Hospital, Montréal, Québec, Canada; Federal University of Rio de Janeiro, Brazil

## Abstract

**Objectives:**

Increasingly, medical research involves patients who complete outcomes in different languages. This occurs in countries with more than one common language, such as Canada (French/English) or the United States (Spanish/English), as well as in international multi-centre collaborations, which are utilized frequently in rare diseases such as systemic sclerosis (SSc). In order to pool or compare outcomes, instruments should be measurement equivalent (invariant) across cultural or linguistic groups. This study provides an example of how to assess cross-language measurement equivalence by comparing the Center for Epidemiologic Studies Depression (CES-D) scale between English-speaking Canadian and Dutch SSc patients.

**Methods:**

The CES-D was completed by 922 English-speaking Canadian and 213 Dutch SSc patients. Confirmatory factor analysis (CFA) was used to assess the factor structure in both samples. The Multiple-Indicator Multiple-Cause (MIMIC) model was utilized to assess the amount of differential item functioning (DIF).

**Results:**

A two-factor model (positive and negative affect) showed excellent fit in both samples. Statistically significant, but small-magnitude, DIF was found for 3 of 20 items on the CES-D. The English-speaking Canadian sample endorsed more feeling-related symptoms, whereas the Dutch sample endorsed more somatic/retarded activity symptoms. The overall estimate in depression scores between English and Dutch was not influenced substantively by DIF.

**Conclusions:**

CES-D scores from English-speaking Canadian and Dutch SSc patients can be compared and pooled without concern that measurement differences may substantively influence results. The importance of assessing cross-language measurement equivalence in rheumatology studies prior to pooling outcomes obtained in different languages should be emphasized.

## Introduction

Health-related patient-reported outcome (HR-PRO) measures assess patient health, well-being, and response to treatment based on patient perspectives. They may reflect complex constructs, such as health-related quality of life, or narrower constructs, such as individual symptoms (e.g., pain or fatigue) that are used to assess health status in patients with rheumatic diseases [1]–[4]. Growing recognition of the importance of HR-PROs and their increasing integration into both research and clinical practice has led to initiatives to improve their operationalization.

In the rheumatic diseases, OMERACT (Outcome Measures in Rheumatology) [5] has delineated a set of standards by which measures can be evaluated, including the truth or validity, discrimination, and feasibility of measures. Recently, the COSMIN checklist (Consensus-based Standards for the selection of health status Measurement Instruments) [6] was developed to establish criteria for evaluating the methodological quality of studies on HR-PROs. In addition to the standards described by OMERACT, the COSMIN checklist emphasizes the importance of establishing the cross-cultural validity of HR-PROs.

The cross-cultural validity of HR-PROs is increasingly important in medical research, since patients who complete outcome measures in different languages are commonly included in the same study. For instance, this often occurs in countries with more than one highly common language, such as Canada (French/English) or the United States (Spanish/English). In addition, multicenter trials that include centres from different countries are increasingly frequent. Particularly in rare diseases, such as systemic sclerosis (SSc), effective research often requires international collaboration to include a sufficient number of patients for adequately powered studies. The Scleroderma Clinical Trials Consortium [7] and the EULAR Scleroderma Trials and Research group [8], for instance, routinely conduct multicentre drug trials involving patients from multiple countries and measures translated into multiple languages. Recently, the Scleroderma Patient-centered Intervention Network was organized to test psychosocial and rehabilitation interventions in patients from across Europe and North America [9].

As described in the COSMIN checklist [6], it is important to assess the degree to which outcome measures generate scores that are equivalent or invariant across linguistic or cultural groups, meaning that individuals from different groups with similar levels of an outcome of interest should obtain equal scores on the measure and respond similarly to individual items of the measure. This is because differences in the meaning of items due to translation or cultural differences in item interpretation can lead to responses that differ across groups even when levels of the outcome being measured are similar. Measurement differences between translated questionnaires can be a serious threat to the validity of cross-cultural comparisons, because when measures are not equivalent metrically, it is not possible to determine if any observed differences between groups reflect real differences or are a consequence of measurement artifacts (e.g., linguistic/cultural differences) [10]. Therefore, cross-cultural validity should be established if HR-PROs are to be pooled among study participants from different countries or used to compare results between patients from different cultural or linguistic groups [11].

Differential item functioning (DIF) is said to occur when an item of a HR-PRO has different measurement properties for one group compared to another, irrespective of true differences of the construct measured. Diverse statistical methods for assessing the presence of DIF are available, based on non-parametric, parametric or latent variable models, each with its own advantages and disadvantages [12]. Generally, however, the presence of DIF is assessed by identifying differences in individual item scores across groups that are present even after controlling for levels of the overall construct being measured. When DIF is identified, it is assumed that scores on the item are influenced by group characteristics that are not directly related to the construct being measured. When translated versions of HR-PROs are administered in different cultural settings, DIF may occur because of alterations in item meaning due to translation or because of cultural factors that influence interpretation of item meaning.

HR-PRO measures for depressive symptoms are increasingly used among patients with chronic medical illness [13], [14]. This is also the case for patients with SSc, which is an autoimmune disease characterized by thickening of the skin as a result of fibrosis, as well as involvement of multiple internal organs, most commonly the lungs, gastrointestinal tract and heart [15]. In SSc, the Center for Epidemiologic Studies Depression Scale (CES-D) [16] has been used to assess depressive symptoms in English [17]–[20], French [19], [20], Dutch [21], and German [22]. The CES-D was originally developed in the USA to measure depressive symptomatology in the general population [16]. The scale has also shown to be a reliable and valid measure of depressive symptoms across various patient samples, including SSc [23]. No studies, however, in any patient group have assessed the degree to which translated versions of the CES-D are measurement equivalent versus exhibiting substantive DIF, possibly due to the unfamiliarity of researchers and clinicians with the need for assessment of cross-language measurement properties or the methods by which this can be done. This study provides an example of how to assess cross-language measurement equivalence by comparing the CES-D between English-speaking Canadian and Dutch SSc patients.

## Methods

### Ethics Statement

The English-speaking sample of this cross-sectional study consisted of patients with SSc enrolled in the Canadian Scleroderma Research Group Registry (CSRG). The study was approved by the Institutional Review Board of McGill University. The Dutch sample consisted of patients with SSc enrolled in a 3-year cohort study in Nijmegen, The Netherlands. The study was approved by the Institutional Review Board of the Radboud University Medical Center Nijmegen (CMO2008/109). All patients provided written consent for their information to be stored in a computer database and used for research.

### Patients and Procedures

#### English-speaking sample

The English-speaking sample consisted of patients enrolled in the CSRG who completed the CES-D in English from September 2004 through April 2011. Patients in the Registry are recruited from 15 centers across Canada. To be eligible for the Registry, patients must have a diagnosis of SSc confirmed by a Registry rheumatologist, be 18 years of age, and be fluent in English or French. Registry patients undergo extensive physical evaluation at annual visits and complete a series of self-report questionnaires in their preferred language (English or French). For patients who completed the CES-D at multiple annual visits, only data from the most recent visit was included in analyses in the present study.

#### Dutch sample

The Dutch sample consisted of SSc patients treated at the Sint Maartenskliniek or Radboud University Medical Center Nijmegen, The Netherlands who completed the baseline assessment of a 3-year cohort study, including the CES-D in Dutch, between June 2008 and February 2010. To be eligible, patients had to have a diagnosis of SSc according to the preliminary American College of Rheumatology classification criteria [24]. Exclusion criteria for participation in the cohort were a life expectancy <1 year, acute serious complications (e.g., renal crisis), severe psychiatric co-morbidity, other serious co-morbidities (e.g., cancer) and insufficient knowledge of the Dutch language.

### Measures

#### Demographics and disease characteristics

Demographic variables for both samples included age, sex, marital status, education and current employment status. Disease characteristics were assessed by study rheumatologists in both samples, including disease duration, SSc subtype, and the modified Rodnan skin score (mRSS). Disease duration was defined as time since onset from first non-Raynaud symptom. Patients were classified as having limited or diffuse SSc. Limited SSc was defined as skin involvement distal to the elbows and knees only, whereas diffuse SSc was defined as skin involvement proximal to the elbows and knees, and the trunk also [25]. The mRSS is a standardized rating of skin involvement ranging from 0 (no involvement) to 3 (severe thickening) in 17 body areas (total score range 0–51) [26].

#### Symptoms of depression

The CES-D [16] is a 20-item measure that assesses the frequency of symptoms during the past week on a 0–3 Likert scale (“rarely or none of the time” to “most or all of the time”). Standard cutoffs are ≥16 for “possible depression” and ≥23 for “probable depression” [16]. A cutoff of ≥19 has been suggested in arthritis [27]. The CES-D used in the English-speaking sample was the original version [16], which has shown to be a reliable and valid measure of depressive symptoms in patients with SSc [23]. In the Dutch sample, the original translation [28], which has been shown to be reliable and valid across diverse settings was used.

### Statistical Analyses

Demographics and disease characteristics were compared between the English-speaking and Dutch samples using the chi-square statistic for categorical variables and t-tests for continuous variables.

**Figure 1:**
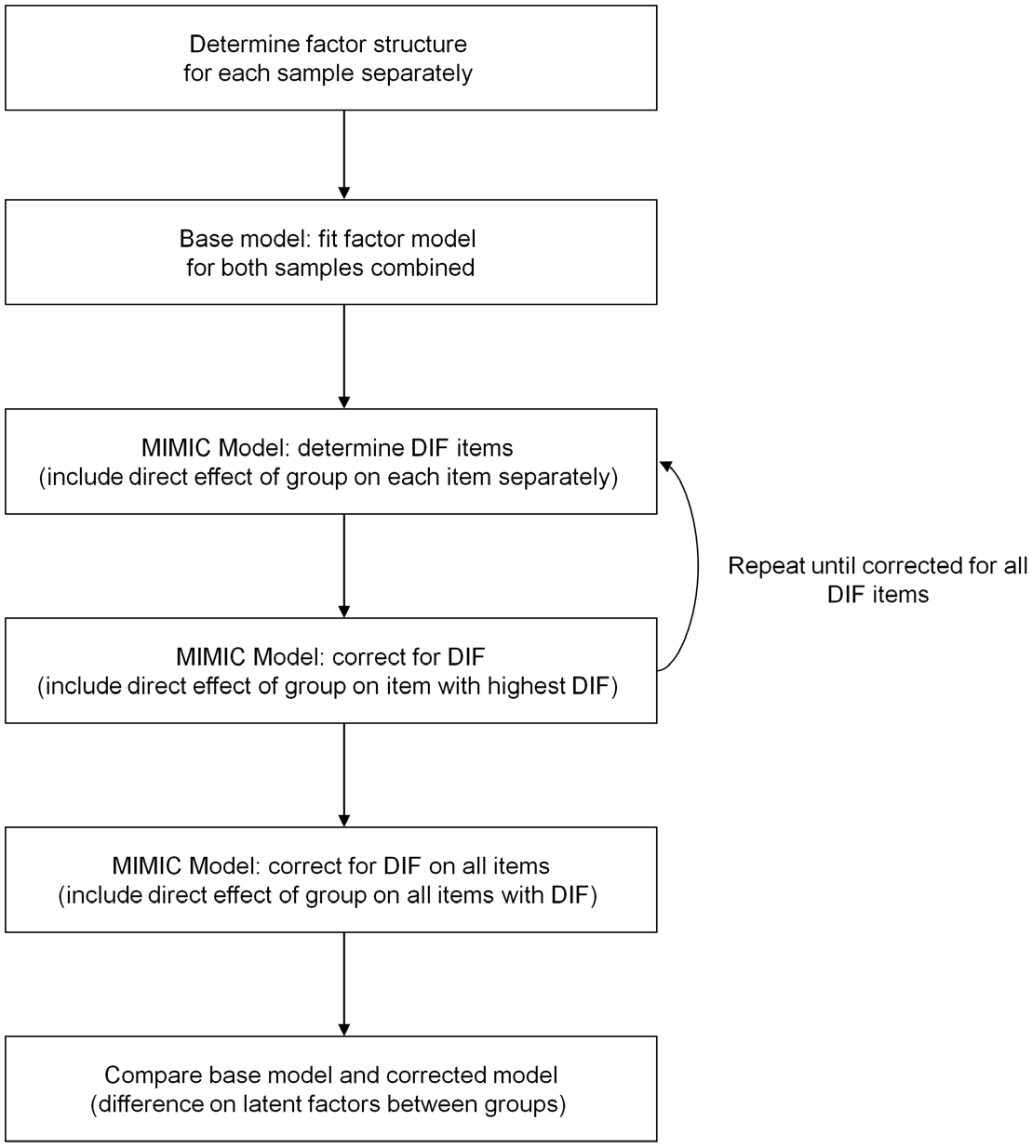
Flowchart of steps to be undertaken in DIF analysis.

A flowchart of steps undertaken in the DIF analysis is depicted in Figure 1. First, the factor structure of the CES-D was assessed for each sample separately using confirmatory factor analysis (CFA). Ideally for DIF assessment, the simplest structure with reasonable fit will be used. Thus, an initial CFA model was constructed with Mplus [29] to determine if a single-dimensional structure of the CES-D in SSc could be reasonably used in the DIF analysis versus an alternative structure. Selection of an alternative structure was based on a previous validation study of the CES-D in SSc [23]. Item responses for the CES-D were ordinal Likert data, so the weighted least squares estimator with a diagonal weight matrix, robust standard errors, and a mean- and variance-adjusted chi-square statistic was used with delta parameterization [29]. Modification indices were used to identify pairs of items within scales for which model fit would improve if error estimates were freed to covary and for which there appeared to be theoretically justifiable shared method effects (e.g., similar wording) [30]. To assess model fit, the chi-square test, the Tucker-Lewis Index (TLI) [31], the Comparative Fit Index (CFI) [32] and the Root Mean Square Error of Approximation (RMSEA) [33] were used. Since the chi-square test is highly sensitive to sample size, it can lead to the rejection of well-fitting models [34]. Therefore, the TLI, CFI and RMSEA fit indices were emphasized. Good fitting models are indicated by a TLI and CFI≥0.95 and RMSEA≤0.06 [35]. Once the factor structure was established for each sample separately, a CFA model was fit that included patients from both samples combined.

**Figure 2:**
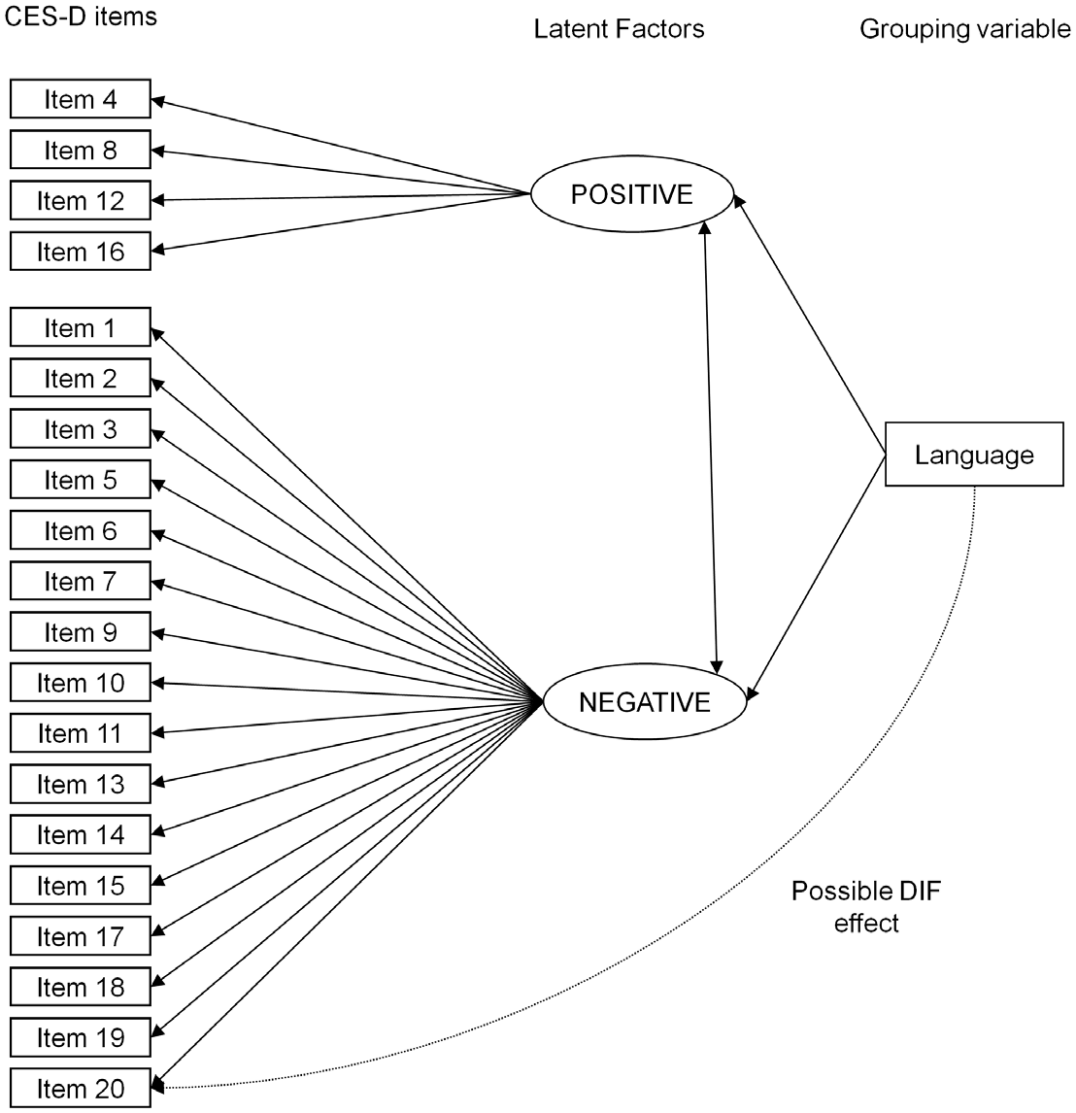
The MIMIC Model for the Centre for Epidemiologic Studies Depression Scale (CES-D).

The Multiple-Indicator Multiple-Cause (MIMIC) model was utilized to determine if items of the CES-D exhibited DIF for English-speaking versus Dutch patients. MIMIC models for DIF assessment are based on structural equation models, in which the group variable (English/Dutch) is added to the basic CFA model as an observed variable. Thus, the base MIMIC model consists of the CFA factor model with the additional direct effect of group on the latent factors, which serves to control for group differences on the level of the latent factors. Since there were statistically significant difference between our samples, we also controlled for demographic and disease variables (age, sex, marital status, education, current employment status, SSc subtype, mRSS and disease duration) by adding a direct effect of these variables on the latent factors, Then, to assess potential DIF, the direct effect of group on CES-D items is assessed for each item separately, by regressing the items, one at a time, on group (see Figure 2). Each item is tested separately to determine if there is statistically significant DIF, represented by a statistically significant link in the model from group to the item, after controlling for any differences in the overall level of the latent factor between groups. If there is DIF for one or more items, the item with the largest magnitude of DIF is considered to have DIF, and the link between the linguistic group variable and that item is included in the model. Then, this procedure is repeated until none of the remaining items show significant DIF. Once all items with significant DIF are identified, the potential magnitude of DIF items collectively, identified via assessment of statistical significance, can be evaluated by comparing the difference on the latent factor between groups in the baseline CFA model and after controlling for DIF. Since the CES-D consists of a large number of items, Hommels’ [36] correction for multiple testing was applied. CFA and DIF analyses were conducted using Mplus [29], all other analyses were conducted using Stata/IC 10.1 (StataCorp LP, College Station, TX).

## Results

### Sample Characteristics

Demographics and disease characteristics for both samples are displayed in Table 1.

**Table 1:** Demographic and medical characteristics for both SSc samples.

Variable	English-speaking (N = 922)	Dutch (N = 213)	P value
Female (%)	782 (84.8)	144 (67.6)	<0.001
Mean age, years (SD)	55.2 (12.3)^a^	56.4 (12.0)	0.17
Higher education (% >12 years)	451 (49.2)^b^	86 (41.1)^c^	0.04
Currently working (%)	383 (41.7)^d^	70 (32.9)	0.02
Married or living as married (%)	764 (82.9)	161 (75.6)	0.01
Limited disease (%)	556 (60.3)	157 (75.1)^c^	<0.001
Mean disease duration (SD)	11.1 (9.3)^e^	9.2 (8.0)^f^	0.01
Mean modified Rodnan Skin Score (SD)	10.5 (9.6)^g^	6.4 (6.0)^h^	<0.001
CES-D score, mean (SD)	14.3 (10.3)	12.8 (9.6)	0.05

Due to missing values: ^a^N = 920, ^b^N = 918, ^c^N = 209, ^d^N = 919, ^e^N = 879, ^f^N = 206,^ g^N = 905,^ h^N = 207.

#### English-speaking sample

In total, 976 patients completed the CES-D in English. Six patients were excluded from analysis, because they had >2 missing values on the CES-D. Furthermore, 48 patients were excluded because they were diagnosed with sine SSc, but not diffuse or limited SSc. Of the remaining 922 patients, 84.8% were female. Most patients (82.9%) were married or cohabitating. The mean CES-D score was 14.3 (SD = 10.3) and the percentage of patients scoring ≥16 was 37.7%. The percentage of patients with CES-D≥19 was 28.7%.

#### Dutch sample

In total, 215 patients completed the baseline questionnaires. Two patients were excluded from the analysis because they had >2 missing values on the CES-D. Of the 213 patients in the sample, 67.6% were female. Most patients (75.6%) were married or cohabitating. The mean CES-D score was 12.8 (SD = 9.6) and the percentage of patients scoring ≥16 was 31.9%. The percentage of patients with CES-D≥19 was 24.9%.

Compared with the English-speaking sample, patients in the Dutch sample were significantly more likely to be male and to have limited disease. They were less likely to have completed more than 12 years of education, or to be currently working. Furthermore, patients in the Dutch sample had significantly shorter disease duration and lower mean mRSS scores. Mean CES-D scores in the Dutch sample were somewhat lower than in the English-speaking sample (P = 0.05). The proportion of patients with CES-D≥16 (P = 0.11) and CES-D ≥19 (P = 0.27) did not differ significantly.

### Confirmatory Factor Analysis

For both samples, a single-factor structure was assessed initially. In both the English-speaking and Dutch samples, the fit was poor (English-speaking: χ^2^(76) = 2218.8, P<0.001, CFI = 0.71, TLI = 0.88, RMSEA = 0.18; Dutch: χ^2^(50) = 259.0, P<0.001, CFI = 0.87, TLI = 0.94, RMSEA = 0.14). Inspection of the modification indices for both samples indicated correlated error terms of all positively worded items (items 4, 8, 12 and 16). Since allowing the error terms of these items to be correlated with each other would essentially result in specifying a second factor, a two-factor model was refitted, with two correlated factors: positive and negative [27]. The two-factor model showed good fit to the data in both samples (English-speaking: χ^2^(81) = 572.6, P<0.001, CFI = 0.93, TLI = 0.98, RMSEA = 0.08; Dutch: χ^2^(51) = 128.3, P<0.001, CFI = 0.95, TLI = 0.98, RMSEA = 0.08). In both samples, inspection of modification indices indicated that freeing error terms to covary for items 15 and 19, 17 and 18, and 7 and 20, would improve model fit, and in each case there was clearly recognizable overlap in the items’ content. Therefore, the model was refitted to the data, allowing the error terms for those items to be correlated. This change resulted in a model with excellent fit to the data in both samples (English-speaking: χ^2^(81) = 345.1, P<0.0001, CFI = 0.96, TLI = 0.99, RMSEA = 0.06; Dutch: χ^2^(51) = 94.6, P<0.0001, CFI = 0.97, TLI = 0.99, RMSEA = 0.06).

### Differential Item Functioning

The two-factor model that was fit for each sample individually was fit with all patients in the same model (Table 2), along with a direct effect of group (English/Dutch) on both factors (“positive”/“negative”). As shown in Table 3, model fit for the combined sample for this base model was excellent. Prior to accounting for DIF, English-speaking patients had higher latent factor scores than Dutch patients: 0.19 standard deviations for “positive” factor scores, and 0.03 standard deviations for “negative” factor scores, although neither difference was statistically significant. Initially, three items showed significant DIF: items 3, 4, and 7. Item 3 (z = 4.4, P<0.001) and item 4 (z = 4.4, P<0.001) had higher scores in the English-speaking sample, controlling for differences on the latent factors. On the other hand, item 7 (z = −3.6, P<0.001) had higher scores in the Dutch sample. All three items continued showing DIF, throughout the sequence of correcting for DIF on the other items.

**Table 2:** Factor loadings on the “positive” and “negative” factors of the CES-D.

	Base model^a^		DIF corrected model^b^	
	Factor loading	95% Confidence Interval	Factor loading	95% Confidence Interval
**Positive factor items:**				
4. Good	0.57	[0.51, 0.62]	0.56	[0.50, 0.62]
8. Hopeful	0.74	[0.70, 0.78]	0.74	[0.70, 0.78]
12. Happy	0.91	[0.87, 0.95]	0.91	[0.86, 0.95]
16. Enjoy	0.83	[0.79, 0.87]	0.83	[0.79, 0.87]
**Negative factor items:**				
1. Bothered	0.74	[0.71, 0.78]	0.74	[0.71, 0.78]
2. Appetite	0.51	[0.46, 0.57]	0.51	[0.46, 0.57]
3. Blues	0.86	[0.83, 0.89]	0.86	[0.83, 0.89]
5. Mind	0.71	[0.68, 0.76]	0.71	[0.67, 0.75]
6. Depressed	0.90	[0.88, 0.92]	0.90	[0.88, 0.92]
7. Effort	0.67	[0.63, 0.71]	0.67	[0.63, 0.71]
9. Failure	0.77	[0.72, 0.81]	0.77	[0.72, 0.81]
10. Fearful	0.68	[0.63, 0.72]	0.68	[0.63, 0.71]
11. Sleep	0.47	[0.41, 0.52]	0.47	[0.41, 0.52]
13. Talk	0.70	[0.66, 0.74]	0.70	[0.66, 0.74]
14. Lonely	0.77	[0.74, 0.81]	0.77	[0.74, 0.81]
15. Unfriendly	0.49	[0.41, 0.56]	0.49	[0.41, 0.56]
17. Cry	0.73	[0.68, 0.78]	0.73	[0.68, 0.78]
18. Sad	0.85	[0.82, 0.88]	0.85	[0.82, 0.88]
19. Dislike	0.68	[0.61, 0.74]	0.68	[0.61, 0.74]
20. Get going	0.69	[0.65, 0.73]	0.69	[0.65, 0.73]
**Correlation of positive and negative latent factors:**	0.47	[0.42, 0.52]	0.47	[0.42, 0.52]

**Table 3:** Factor loadings of DIF items and influence on the overall estimates of depression latent factor scores.

	Base model^a^		DIF corrected model^b^	
	Factor loading	95% Confidence Interval	Factor loading	95% Confidence Interval
**Direct effects on items attributable to English language:**				
Item 3. Blues			0.35	[0.18, 0.51]
Item 4. Good			0.38	[0.21, 0.56]
Item 7. Effort			0.23	[−0.37, −0.09]
**Structural effect of English language on latent factors:**				
Group on positive	0.19	[−0.02, 0.40]	0.09	[−0.11, 0.30]
Group on negative	0.03	[−0.16, 0.22]	0.02	[−0.17, 0.21]
**Model fit summary:**				
Model chi-square (df), P-value	554.49 (166), P<0.001		528.58 (166), P<0.001	
CFI	0.96		0.96	
TLI	0.98		0.98	
RMSEA	0.05		0.05	

a Not corrected for DIF,

b Corrected for DIF for item 3, 4, and 7.

CFI =  Comparative Fit Index; TLI =  Tucker-Lewis Index; RMSEA =  Root Mean Square Error of Approximation.

After correcting for DIF, compared to the base model, there was a decrease of 0.10 standard deviations on the “positive” latent factor and a decrease of 0.01 standard deviations on the “negative” latent factors in the difference between English-speaking and Dutch patients, and confidence intervals were overlapping, as shown in Table 3. Thus, although there was statistically significant DIF on three CES-D items, this did not influence the overall estimates of depression latent factor scores between English-speaking and Dutch patients substantively.

## Discussion

In order to compare or pool data obtained with HR-PRO measures that are administered in different languages, cross-cultural or cross-linguistic equivalence of scores should be established. As an example of how to conduct such type of studies, in the present study the cross-linguistic measurement equivalence was assessed for the CES-D in English-speaking Canadian and Dutch SSc patients. Significant DIF was found for 3 of 20 items on the CES-D. However, the magnitude of DIF for each of these items was very small, and the effect on overall CES-D scores was negligible. This means that if there is DIF, it is so small that CES-D scores would not be influenced meaningfully by it.

Small-magnitude DIF was found for some items in our study. DIF in cross-linguistic comparisons may be caused by a lack of conceptual equivalence due to differences in content, format, difficulty or cultural relevance for the English-speaking compared to the Dutch sample [10]. The Dutch sample scored higher, even after controlling for latent depression symptom levels, on 2 items that were similar in meaning and related to energy levels and effort. Canadian English-speaking patients, on the other hand, appeared to endorse more ‘feeling’ symptoms. It is possible that this is related to cultural differences in how symptoms are experienced or expressed. It is also possible, however, that these differences may be related to translation. For item 7 (“Effort”), no remarkable differences were found in the translations. This was not the case, however, for items 3 (“Blues”) and 4 (“Good”). In fact, there are many examples of discussions in the published literature related to the difficulty of translating from English “feeling blue” and related expressions such as “having the blues” [37]–[39]. In many languages, including Dutch, a strictly lexical translation for these terms is meaningless. Therefore, in translated versions, words need to be found with sufficient similarity to convey the concept, which might lead to slight differences between translated versions. For item 4 (“I felt that I was just as good as other people”), in the Dutch version of the CES-D, the translation of “good” is interpreted as “worth,” which has a slightly different connotation, and, therefore, might have influenced responses differently across groups.

Despite the identification of minor DIF for several items, this study found that CES-D scores for English and Dutch SSc patients with equal levels of depressive symptoms would be expected to be highly similar. In other words, possible DIF on single items was of very small magnitude and had negligible influence on the overall score. Therefore, scores generated with the English and Dutch versions of the CES-D are comparable and do not require adjustment for linguistic differences. This is an important result given the increasingly common use of multinational collaborations to conduct research in rare diseases, such as SSc [7]–[9]. Future studies should extend the current assessment of the CES-D into other languages. In addition, measurement equivalence should also be assessed for other frequently used HR-PROs central to research in rheumatic diseases, including, for instance, the Health Assessment Questionnaire [40] and the SF-36 [41].

There are limitations that should be considered in interpreting the results of this study. Because of the difference in sample size between the samples, the core model used to assess DIF relied more on data from English-speaking patients than Dutch patients. However, since the initial factor analysis yielded the same results in both samples, it does not seem likely that this would have influenced results substantially. A second limitation relates to differences in sample recruitment. Dutch patients were recruited from two hospitals, between 2008 and 2010, whereas the English-speaking patients were recruited from 15 centers from across Canada between 2004 and 2011. Furthermore, there were some differences in inclusion criteria for the two samples and in the demographic (in particular, sex) and disease characteristics (in particular, disease subtype and duration) of the samples. However, the sensitivity analysis correcting for differences in demographics and disease characteristics between samples yielded virtually the same results as the non-corrected model, which suggests that differences in sample characteristics did not likely influence the results. Finally, MIMIC models do not test for non-uniform DIF. Non-uniform DIF means that the amount of DIF is unequal for different levels of the outcome of interest, in our case depression. On the other hand, MIMIC models do allow for adjustment for important covariates that may differ between comparison groups, which is an important strength of the model.

In conclusion, there were 3 CES-D items with evidence of minor DIF between the English and Dutch samples. Overall, however, there was no evidence that these minor differences influenced overall scores. Therefore, CES-D scores from English-speaking Canadian and Dutch SSc patients can be compared and pooled without concern that measurement differences may substantively influence results.

Given the importance of international collaborations and multi-center trials, in particular for research on rare diseases such as SSc, additional studies are needed that assess the measurement of other key HR-PROs across languages. Researchers across areas of research and languages should be aware of the importance of assessing cross-language measurement equivalence of HR-PROs prior to pooling results obtained in different languages.
